# The roles of serotonin in cell adhesion and migration, and cytoskeletal remodeling

**DOI:** 10.1080/19336918.2021.1963574

**Published:** 2021-09-08

**Authors:** Joe Anand Kumar John Jayakumar, Mitradas M. Panicker

**Affiliations:** a Manipal Academy of Higher Education, Manipal, India; b National Centre for Biological Sciences, Tata Institute of Fundamental Research, Bengaluru, India; c Present Address - Department of Physiology and Biophysics, School of Medicine, University of California, Irvine, USA

**Keywords:** Serotonin, cell adhesion and migration, cytoskeletal remodeling, platelet aggregation, immune cells, neuronal cells, development, antipsychotics, antidepressants, homeostasis, covid-19

## Abstract

Serotonin is well known as a neurotransmitter. Its roles in neuronal processes such as learning, memory or cognition are well established, and also in disorders such as depression, schizophrenia, bipolar disorder, and dementia. However, its effects on adhesion and cytoskeletal remodelling which are strongly affected by 5-HT receptors, are not as well studied with some exceptions for e.g. platelet aggregation. Neuronal function is strongly dependent on cell-cell contacts and adhesion-related processes. Therefore the role played by serotonin in psychiatric illness, as well as in the positive and negative effects of neuropsychiatric drugs through cell-related adhesion can be of great significance. In this review, we explore the role of serotonin in some of these aspects based on recent findings.

## Introduction

Serotonin, 5-hydroxytryptamine (5-HT), a monoamine, is involved in a wide range of functions, which include platelet aggregation, cell proliferation, cell transformation, vascular smooth muscle contraction, mood, appetite, cognition, learning and memory, thermoregulation, locomotion, sleep, sexual behavior, endocrine secretion, pain, and immune responses [1–3]. 5-HT, in the animal kingdom, is synthesized from the amino acid tryptophan by tryptophan hydroxylase (TPH) and AADC – an aromatic amino acid decarboxylase though in *Drosophila* and mouse phenylalanine hydroxylase is also known to substitute for TPH [4]. In mammals, more than 90% of the 5-HT present is produced by the enterochromaffin cells in the gastrointestinal tract and the rest is chiefly produced in the CNS by the serotonergic neurons of raphe nuclei present in the brain stem [5]. Apart from endogenous synthesis, 5-HT is supplied to the developing embryos from maternal and placental sources [6]. While 5-HT is not known to be supplied *via* dietary intake or cannot cross the blood-brain barrier, dietary supplementation with tryptophan or 5-hydroxytryptophan an intermediate precursor of 5-HT can raise the blood 5-HT, and since both of these molecules can cross the blood-brain barrier it can increase 5-HT in the brain as well [7]. Moreover, 5-HT is expressed very early in the development in various invertebrates and vertebrates even before the formation of the nervous system and is reported to play a developmental role [8–11].

**Figure 1.**
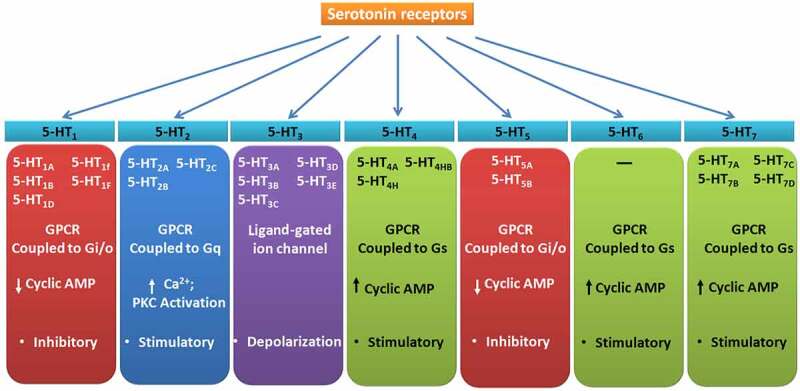
Classification and characteristics of serotonin receptors

5-HT can activate 14 known receptor subtypes (not including splice and edited variants), which are grouped into seven major families (Figure 1) based on their structural and functional similarities [12]. All 5-HT receptors are G-protein-coupled receptors, except those belonging to the 5-HT_3_ family which are ionotropic ligand-gated ion-channels [12].

The distribution and functions of various 5-HT receptors in mammals are highly diverse and briefly noted here. These have been mostly studied in the context of the nervous system. 5-HT_1A_ is present in limbic areas, anterior raphae nuclei, and interpeduncular nucleus, and is implicated in the regulation of the cardiovascular system, neuroendocrine responses such as secretion of adrenocorticotropic hormone, regulation of body temperature, sleep states, mood, and neurogenesis. 5-HT_1A_ knockout mice are seen to exhibit anxiety, depression, and cognitive deficits [12,13]. 5-HT_1B_ is expressed in the hippocampus, striatum, cerebral cortex, cerebellum, and vascular tissues, which is associated with contraction of rat caudal arteries, inhibition of noradrenaline release, inhibition of plasma extravasation, and it plays a role in migraine [12,13]. 5-HT_1D_ present in low levels in the brain is a prime target for antimigraine drugs [12,13]. 5-HT_2A_ is present in the cortex, hippocampus, platelets, vascular smooth muscle cells, and gastrointestinal tract (GIT) is associated with learning and memory, behavior, sexual functions, endocrine functions, thermoregulation, gastrointestinal motility, platelet aggregation, vascular smooth muscle contraction [14,15]. 5-HT_2B,_ present in rat fundus, gut, heart, kidney, lung, cerebellum, lateral septum, dorsal hypothalamus and medial amygdala, is seen to be quintessential for the development of the cardiovascular system [14,16]. 5-HT_2C_ reported in the choroid plexus, hippocampus, amygdala, human cerebral cortex, cerebellum and substantia nigra (SN), endopiriform nuclei, cingulate, and piriform cortex, is seen to play an important role in feeding behavior[17]. 5-HT_3_ receptor family subtypes 5-HT_3A_ and 5-HT_3B_, are expressed in the amygdala, hippocampus, cortex at the CNS, peripheral autonomic ganglion and GIT, and are seen to be associated with the regulation of emetic reflex, intestinal motility and, in addition, has roles in the cardiovascular system [12]. 5-HT_4_ is seen to be present in the olfactory tubercle, islands of Calleja, substantia nigra, ventral pallidum, striatum, septum, hippocampus, amygdala, heart, and GIT. 5-HT_5_ is present in the cerebral cortex, dentate gyrus, pyramidal cell layer within hippocampal fields CA1-3, granule cell layer of the cerebellum, and tufted cells of the olfactory bulb, cerebral cortex, hippocampus, and cerebellum. The 5-HT_5A_ knockout mouse also shows increased exploratory activity in a novel environment. 5-HT_6_ is found in the striatum, nucleus accumbens, olfactory tubercle, and cortex, is moderately expressed in the amygdala, hypothalamus, thalamus, cerebellum, and hippocampus, corpus striatum, nucleus accumbens, Islands of Calleja, olfactory tubercle, and the choroid plexus. Moderate levels are found in the hippocampal formation and cerebral cortex, thalamus, hypothalamus, and substantia nigra and is involved in various functions such as cognition, learning and memory and Alzheimer’s disease [18,19]. 5-HT_7_ is present in the ileum, spleen, endocrine glands, arteries, thalamus, hypothalamus, cerebral cortex, hippocampus, and amygdala [20]. It is seen to be involved in the regulation of the endocrine system, the circadian rhythm, and temperature regulation, sleep, neuropsychiatric disorders, memory and learning, locomotor functions, migraine pain, substance abuse, respiratory, cardiovascular and intestinal systems [21,22].

Serotonin and its receptors are expressed at both central nervous system and periphery and are known to modulate many functions, however, its lesser known roles in cell adhesion, migration, or cytoskeletal remodeling are recently gaining interest. The adhesion-related processes associated with the serotonergic system reviewed here are as follows. Serotonin is classically associated with platelet aggregation and inhibitors of 5-HT_2A_ are clinically used as drugs to prevent platelet aggregation. Serotonin and its receptors are also known to play a role in adhesion, migration and proliferation of vascular smooth muscle cells and pathogenesis of atherosclerosis. The adhesion-related processes mediated by serotonin in immune system are observed in mast cells, eosinophils, dendritic cells etc., for e.g in cell adhesion, migration, cytoskeletal remodeling and cell shape. Serotonin is known to augment wound healing, which is seen to be relevant for its role in fibrosis also, which is characterized by excessive extracellular matrix protein secretion. In the central nervous system, serotonin has obvious roles in the expression and modulation of adhesion molecules including NCAM, synaptic adhesion and neurite remodeling. The role of serotonin in cell adhesion is also seen in developmental processes for e.g. in gastrulation and interneuron migration. As reviewed here, adhesion-related roles of serotonergic system are interlinked with neuropsychiatric disorders and their medication.

## Examples of serotonin-mediated adhesion, migration, and remodeling of the cytoskeleton

**Figure 2.**
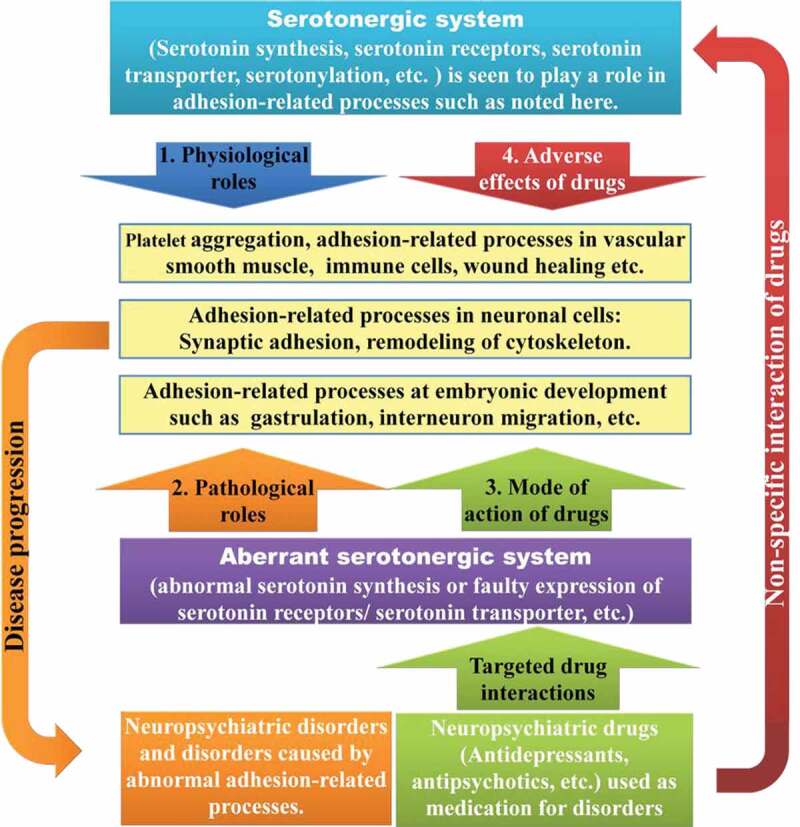
The schematic representation of the adhesion-related effects mediated by the serotonergic system. The link between the serotonergic system and adhesion-related processes can be depicted as follows. (1) Physiological roles: The adhesion-related processes (yellow) – platelets, vascular smooth muscle cells, immune cells, wound healing, neuronal cells, in development, etc., could be modulated by serotonergic system as its physiological role (blue). (2) Pathological roles: An aberrant serotonergic system for e.g. abnormal levels of expression of serotonin, serotonin receptors or transporter, etc., (purple) could also affect the adhesion-related processes, and such abnormal adhesion-related processes could cause the disease progression of neuropsychiatric disorders (orange). (3) Mode of action of drugs: The neuropsychiatric drugs targeting the aberrant serotonergic system could evoke its desired response of mitigating neuropsychiatric symptoms by modulating the adhesion-related processes (green). (4) Adverse effects of drugs: The neuropsychiatric drugs can also act nonspecifically on other components of serotonergic system and result in off-target adverse effects (brown)

Though the serotonergic system is mostly associated with the central nervous system and primarily recognized in neuronal signaling, emerging roles at the periphery in lesser known cellular processes related to adhesion have begun to unravel entirely novel functions. One of the well-known functions of 5-HT related to adhesion is platelet aggregation. In the neuronal context, it has also been speculated that many antidepressants and antipsychotics could mediate their roles by modulating synaptic adhesion molecules and/or the cytoskeleton. Though every detail of these studies is beyond the scope of this review, we aim to analyze in some detail adhesion-related roles of the serotonergic system in all the relevant systems, i.e. platelets, vascular smooth cells, immune cells, neuronal cells and in development, along with its clinical implications (Figure 2). It is important to note that there is a strong correlation between increased platelet aggregation and pathogenesis. For e.g. in COVID-19 patients exhibit increased thrombosis (platelet aggregation) and vascular thromboembolism (circulating platelet clots within the blood vessels) as pathology, which is suggested to have a larger role in disease progression, including multi-organ failure and fatality [23]. The exact mechanism of increased thrombosis in COVID-19 is not fully understood, although it is speculated to be hyper-inflammation (and a subsequent cytokine storm), hypoxia and immobility. As prophylactic anticoagulants such as heparin are administered to prevent COVID-19 associated coagulopathy [24], it would be useful to know if conventional anticoagulants targeting 5-HT_2A_ antagonists such as sarpogrelate or cyproheptadine or antagonists of 5-HT_2_ receptors could be employed for reducing the hyper-coagulability complication in COVID-19 [25].

### Platelet aggregation and migration of vascular smooth muscle cells

Two classical functions of 5-HT also to be discovered very early on were platelet aggregation and vascular smooth muscle contraction, in part due to its initial isolation and identification from fractionated serum [26,27]. Further work established that 5-HT is synthesized and secreted into the blood by enterochromaffin cells and is subsequently taken up and stored in platelets as dense granules [28]. During vascular injury, the contact of platelets with the damaged and exposed vessel walls triggers its aggregation and release of 5-HT. The released 5-HT further stimulates 5-HT_2A_ on platelets and vascular smooth muscle cells, causing amplification of platelet aggregation and vasoconstriction, with resultant clot formation and hemostasis [29,30]. Hence, many conventional antiplatelet aggregation drugs are seen to be inhibitors of 5-HT_2A_, such as sarpogrelate, cyproheptadine, pizotifen, which are used in major occlusive disorders, such as atherosclerosis [31,32]. While the atherosclerotic plaques trigger the first step of pathogenesis i.e. uncontrolled platelet aggregation or thrombosis [33], the associated 5-HT release also initiates a second step, the migration and proliferation of smooth muscle cells into the intima causing thickening of the vessel wall [34]. The migratory effects of 5-HT on smooth muscle cells have been experimentally demonstrated in cultured rat aortic smooth muscle cells [35] and bovine pulmonary artery smooth muscle cells [36] as well, and 5-HT_2_ and 5-HT_4_ receptors were seen to be involved respectively. Progress in atherosclerosis research has now shed light on 5-HT_2A_ as a significant player and potential target for drug discovery [32].

### Adhesion, migration and cytoskeletal remodeling of immune cells

In the immune system, cell adhesion and migration are essential for various functions such as extravasation, chemotaxis, phagocytosis, antigen presentation, secretion of migratory cytokines & extracellular matrices [37], and 5-HT can affect many of these functions [38]. It also turns out that 5-HT plays significant adhesion-related roles in various immune cells, i.e. mast cells, eosinophils, and dendritic cells.

In mice bone marrow-derived mast cells and human CD34+ derived mast cells, 5-HT is seen to cause increased *in vitro* adhesion, migration and actin polymerization, and are dependent on 5-HT_1A_ expressed in these cells. Bone marrow-derived mast cells from 5-HT_1A_-/- mice did not exhibit any of this increased adhesion or actin polymerization, and similarly, in wild type mice, pharmacological inhibition of 5-HT_1A_ abolished the serotonin-mediated increase in the adhesion of these cells to fibronectin substrates. Moreover, intradermal injection of 5-HT also caused migration and accumulation of mast cells to the site of injection in wild type mice, but not in 5-HT_1A_-/- mice [39]. Mast cells are a source of 5-HT [39] and are known to cross the blood-brain barrier and release 5-HT that played a role in learning and memory [40]. Similarly its also known that mast cell-deficient C57BL/6 ^W sh/sh^ mice show impaired spatial learning and memory [41]. So mastocytosis, a condition caused by increased proliferation of mast cells that eventually accumulates in various organs such as skin, liver, etc., is associated with neuropsychiatric disorders such as depression and post-traumatic stress disorder (PTSD) [42].

5-HT is also known to act as a chemo-attractant for eosinophils [43] and causes 5-HT_2A_ dependent *in vitro* migration of human eosinophils, and *in vivo* rolling and migration of murine bone marrow-derived eosinophils within inflamed post-capillary venules [44].

Another role of 5-HT mediated adhesion in the immune system is on dendritic cells, where 5-HT is seen to promote *in vitro* and *in vivo* adhesion, migration, and cytoskeletal modulation. It is seen that 5-HT aids the migration of lung-derived dendritic cells in response to intratracheally injected FITC-labeled OVA, to crossing into mediastinal lymph nodes through epithelial tight junction barriers. Similarly, transwell migration of dendritic cells was seen to be mediated by 5-HT_1B_ and 5-HT_2A_ [45]. In colon explants, treatment with 5-HT_7_ inhibitor caused reduced and diffused migration of dendritic cells, compared to straight and long-distance migration seen with controls. 5-HT_7_ activation also caused actin-mediated extensive morphological changes, *in vitro* transwell migration, and 3D collagen gel invasion of dendritic cells [46].

Moreover, it is seen with numerous studies, that 5-HT is significantly involved in immune disorders such as asthma, wheezing, allergic rhinitis, chronic pulmonary obstructive disease, arthritis, irritable bowel syndrome, Raynaud’s phenomenon/scleroderma or psoriasis [47–52] and the underlying cause or mechanism could be based on adhesion-mediated effects.

A physiological process that is at the intersection of serotonin, platelets and immune cells is wound healing, and serotonin may have an augmentative role. Serotonin is seen to promote cell migration, proliferation, survival, and antiapoptotic effects in keratinocytes and fibroblasts [53]. Moreover, numerous lines of evidence also suggest that autologous platelets enhance healing of skin wounds in humans [54]. Platelets are significant reservoirs for serotonin, and the mechanism could be serotonin-mediated adhesion and migration of cells involved in tissue repair. Yet another role for serotonin is an increased risk of fibrosis with increased blood serotonin, and inhibitors of serotonin receptors such as 5-HT_2A_ and 5-HT_2B_ play protective roles [55,56].

### Modulation of adhesion molecules and cytoskeleton of neuronal cells

The serotonergic system being an integral part of CNS, is known to have significant roles in neuropsychiatric disorders. Several medications are therefore targeted to 5-HT receptors. Recent results from our laboratory suggest that some of the clinically used antipsychotics may modulate adhesion-related processes and F-actin remodeling[57]. Many neuropsychiatric studies, including postmortem brain analysis of schizophrenic patients, have shown expression of synaptic adhesion molecules such as neural cell adhesion molecule (NCAM) to be significantly reduced [58]. The significance of NCAM is even more evident in its relevance in schizophrenia mouse models, such as NCAM1 null mice [59] and in the maternal deprivation mouse model [60] also NCAM expression is seen to be reduced [61]. The modulation of NCAM by 5-HT is driven by the addition of polysialic acid. It is known that non-polysialylated NCAMs are associated with robust and rigid adhesion, polysialylation decreases its adhesiveness and enables dendritic arborization, neuronal migration and synaptic plasticity [62]. Notably, in conditions of depression, in schizophrenic individuals or animal models, polysialylated-NCAMs are also seen to be decreased [63], and antipsychotics or antidepressants used in their treatment promote polysialylation of NCAM [64].

Secondly, remodeling cytoskeleton is integral to neuronal events such as spinogenesis, axonal guidance, growth cone or neurite maturation, synaptogenesis, and plasticity [65]. For these functions, 5-HT is generally seen to have an augmentative role, for e.g. treatment with fluoxetine and vortioxetine, which increases 5-HT concentrations in the synapse, is seen to promote spine enlargement, synaptic contacts and dendritic density [66,67]. Nevertheless, the effects of individual 5-HT receptors are highly variable, based on the receptor type, site of expression, and/or time. For e.g. in rat embryonic neuron culture, 5-HT_1A_ is seen to increase dendritic filopodia density and 5-HT_2A/2C_ is seen to increase the puncta and spine density on embryonic day 11, but on embryonic day 15 they are seen to negate each other’s effects [68]. In another study 5-HT_1A_ has been reported to restrict dendritic growth cone formation [69] while it has also been shown to increase spinogenesis in a similar context [70]. Moreover, 5-HT_2A_ [68,71–73], and 5-HT_7_^46^ [74–77], has been reportedly seen to cause neurite elongation, spinogenesis and synaptogenesis, while 5-HT_3_ is believed to cause a decrease in total axon length and dendritic branching in cultured neurons [78].

### Adhesion and migration in embryonic development

In invertebrates, such as sea urchins, mollusks, starfish, planaria and *Drosophila*, 5-HT is expressed early in the development and is ascribed various pre-nervous roles [8,11,79–82]. In mammals, 5-HT is expressed at different time points in development, for e.g. in rodents 5-HT is expressed in preimplantation embryos and embryonic stem cells [9,83] and in primates it is shown to be present at least from the first month of gestation [84].

Development is a process crucially dependent on differential adhesion, and 5-HT and its receptors play significant roles, which are directly or indirectly related to adhesion. For e.g. HToin *Drosophila*, 5-HT_2Dro_, is seen to play a very important role in its gastrulae, where 5-HT_2Dro_ knockout causes lethally abnormal ectoderm extension due to aberrant adherence junctions, while disruption of 5-HT synthesis also results in similar condition [81,85]. Curiously, mice that lack TPH1 and TPH2 enzymes (involved in the synthesis of 5-HT in the periphery and CNS respectively) are quite normal at birth but does show retarded development initially, and then recovers [86,87]. Such retarded growth could also be attributed to highly deficient maternal care exhibited by dams that lack serotonin [88,89]. It is interesting to note that in mice that lack TPH1 and TPH2 there does not seem to be a total loss of serotonin [90]. One of the possible reasons could be Phenylalanine hydroxylase taking the role of TPH1 in converting tryptophan to 5-hydroxytryptophan the rate-limiting step of serotonin synthesis [4]. 5-HT receptors when globally knocked out, are seen to exhibit a fairly robust survival also suggesting redundancy of functions among 5-HT receptors, which seem to obviate the adverse effects due to absence of individual receptors [10]. These observations suggest that the serotonergic system in development is highly buffered, that even when serotonin synthesis in the developing embryo is inadequate or individual 5-HT receptors are absent, there is some level of compensation. Curiously, when whole embryo cultures were exposed to pharmacological agents, such as inhibitors of SERT, 5-HT_1A_, 5-HT_1B_, 5-HT_1D_ or 5-HT_2A,_ 5-HT_2B,_ or 5-HT_2C_, severe embryonic malformations were observed, and some of them seem to be directly related to adhesion and/or migration [16,91–95]. Moreover, in humans also antipsychotics or antidepressants at pregnancy are seen to be associated with an increased risk of spontaneous abortions, stillbirths or malformations [96,97].

Another adhesion-related process during development, i.e. interneuron migration is also seen to be significantly regulated by 5-HT receptors. 5-HT_3A_ expressed in caudal ganglionic eminence-derived interneurons, plays a role in their migration and laminar positioning to specified cortical plates [98,99]. 5-HT_6_ is also known to affect migration of PC12 and neuronal cells [100–102], and is present in the proliferative zones i.e. subventricular zones (SVZ) and intermediate zone (IZ) of developing mice embryos, and aids interneuron migration [103,104]. Interneuron migration is very important for the spatiotemporal formation of brain regions for e.g. laminar positioning of cortex and formation of neuronal circuits [105,106,107–108]. It is known that faulty interneuron migration results in neuronal migration disorders (National Institute of Neurological Disorders and Stroke information page), which could lead to neuropsychiatric disorders such as autism and schizophrenia. One could perhaps also speculate on interneuron connectivity being affected by 5-HT-mediated adhesion.

## The clinical implication of an aberrant serotonergic system and adverse effects pertinent to cell adhesion and migration, and cytoskeletal remodeling

The role of 5-HT in the central nervous system and neurological disorders such as schizophrenia, anxiety, autism, depression, and bipolar disorders are well known [107–109], and so are the roles of antidepressant and antipsychotic drugs known to effectively relieve neuropsychiatric complaints [110,111]. However, the other roles that the serotonergic system is involved in, especially at the periphery, and in events such as adhesion, migration, and cytoskeletal remodeling have only received moderate attention, despite its significant clinical implications.

Although 5-HT is perceived to be majorly associated with a large number of functions in the CNS, more than 90% of 5-HT is present at the periphery, and is likely to play significant roles. As the 5-HT receptors are present both at the CNS as well as periphery, many neuropsychiatric drugs targeted to affect the CNS are, not so surprisingly, seen to have off-target adverse effects at the periphery. As expected, serotonin is associated with many disorders at the periphery especially in conditions known to increase 5-HT levels in the blood, such as administration of antidepressants. Platelet aggregation, a serotonin-affected phenomenon, also has a strong correlation with atherosclerosis, fibrosis and psoriasis [33,55,112,113]. In conditions such as mastocytosis, atherosclerosis, pulmonary hypertension, or psoriasis, we also see an associated elevated blood level of 5-HT [39,114,115]. In particular, the significance of 5-HT in cardiac fibrosis became evident, from the infamous use of a weight-loss drug Fen-Phen, where the main anorexine fenfluramine, a serotonin reuptake inhibitor, and an agonist of 5-HT_2B_ and caused fibrosis of the heart valve which led to significant mortality [116,117]. Curiously other serotonin reuptake inhibitors have not been reported to cause a similar valvular defect until now. In pulmonary artery hypertension, there is also an over-expression of SERT [48] and maternally administered SSRIs are known to result in pulmonary hypertension in offsprings [118]. Similarly, an increase or decrease in 5-HT is seen to result in the increase or decrease in bone resorption respectively, and humans on SSRI administration are prone to bone fractures [119]. On the contrary, antipsychotics are seen to be beneficial in all of the aforementioned complications, e.g. in delaying the onset of atherosclerosis and fibrosis and for treating pulmonary hypertension, multiple sclerosis, cystic fibrosis, and psoriasis [32,120–122], but is seen to be counter-effective in bone mineralization [123]. Interestingly, in carcinoid syndrome i.e. cancer of the enterochromaffin cells associated with high secretion of 5-HT, as expected, high levels of metastasis are observed [124]. While 5-HT/SSRIs have been linked to promoting metastasis antipsychotics are seen to inhibit it [125,126].

Thus, we see that the adverse effects of abnormal levels of serotonin or faulty expression of receptors/transporter are not confined to just CNS, but are pervasive across multiple systems in the body. Similarly, neuropsychiatric medications such as SSRIs (antidepressants) or antipsychotics are seen to have huge off-target effects outside the CNS. This is one of the major reasons why neuropsychiatric treatment remains a tightrope walk, with specificity largely remaining elusive, often necessitating ‘risk versus benefit’, and limiting the usage of available drugs. As the mode of action of many antidepressants and antipsychotics at the CNS includes replenishing inadequate synthesis, controlling the excessive production of neurotransmitters or modulation of signaling of receptors [127], many of these drugs require chronic administration. This, unfortunately, paves way for several side effects for e.g. agranulocytosis, extrapyramidal symptoms, dyskinesia, weight gain, etc. [128]. In the case of serotonin syndrome, a life-threatening complication arising from increased serotonergic activity following clinical administration of serotonergic agents, SSRIs and many drugs that affect the serotonin metabolism, exhibit symptoms which are termed as the classical triad of cognitive-behavioral changes, neuromuscular excitability, and autonomic instability [129].

Despite significant advances in neuropsychiatry especially serotonin biology, the adverse effects of medications targeting the serotonergic system remain severe. Hence, the path forward in clinical interventions involving the serotonergic system needs to be holistic and multi-targeted, for gaining specificity to minimize off-target effects. To achieve that, it would be imperative to also unravel the complex links between serotonergic systems and lesser-known cellular processes such as cell adhesion and cytoskeletal remodeling, which could aid in understanding the effects of these drugs thereby design optimal drugs with improved clinical results.

The link between the serotonergic system and adhesion-related processes can be depicted as follows. (1) Physiological roles: The adhesion-related processes (yellow) – platelets, vascular smooth muscle cells, immune cells, wound healing, neuronal cells, in development, etc., could be modulated by serotonergic system as its physiological role (blue). (2) Pathological roles: An aberrant serotonergic system for e.g. abnormal levels of expression of serotonin, serotonin receptors or transporter, etc., (purple) could also affect the adhesion-related processes, and such abnormal adhesion-related processes could cause the disease progression of neuropsychiatric disorders (orange). (3) Mode of action of drugs: The neuropsychiatric drugs targeting the aberrant serotonergic system could evoke its desired response of mitigating neuropsychiatric symptoms by modulating the adhesion-related processes (green). (4) Adverse effects of drugs: The neuropsychiatric drugs can also act nonspecifically on other components of serotonergic system and result in off-target adverse effects (brown).
